# Supplementary material for: Comparison of 27-gauge and 25-gauge vitrectomy in the management of tractional retinal detachment secondary to proliferative diabetic retinopathy
Source: PLoS One. 2021 Mar 25;16(3):e0249139. doi: 10.1371/journal.pone.0249139 (PMC7993849; doi:10.1371/journal.pone.0249139)
Supplement: Attachment — Submitted filename: 2021.01.25 Response to Reviewers.docx [file pone.0249139.s002.docx]

**Response to Reviewers**

We thank all the reviewers for giving us the suggestions and opportunity to submit a revised manuscript and hope that this improved manuscript is acceptable for publication in the journal of *PLOS ONE*®.

**Response to Reviewer # 1:**

1. **Comment:** A retrospective small sample size of 43 patients

**Author response:** Thank you for pointing this out. Because of the improved care of diabetes mellitus in Taiwan, the number of TRD secondary to PDR is decreasing, besides, we only enroll cases of TRD with macular involvement, which explained the limited case number. However, it is beyond denial; this is a weak point of this study, and we had mentioned the paragraph discussing about the limitations of the study.

2. **Comment:** The findings of this study are not novel and there more than a few previous studies to compare 27 gauge to 25-gauge vitrectomy for RRD and TRD.

**Author response:** Most of the previous comparative studies of 27- and 25-gauge vitrectomy were discussing about RRD. I believe there are still some findings in this manuscript that can support us to manage more complicated retinal disease like TRD secondary to PDR by choosing 27-gauge vitrectomy.

3. **Comment:** How patients were randomized for both groups? If there were no randomization how the author explains the similarity between both groups?

**Author response:** Since this is a retrospective study, there was no randomization. The similarity between these two groups were because all the cases of 27-gauge were cases having the surgery after August 2018 (the cases were collected from March 2018 to January 2019), when 27-gauge vitrectomy was available in our hospital, before that, we use 25-gauge vitrectomy for PDR with TRD. Since those patients were all DM with TRD. It is of no surprise patients of this disease had similar background conditions and similar demographic data.

4. **Comment:** Lines 135-137: “There was significantly lower rate of using micro forceps in the 27-gauge group (p = 0.004), and a trend toward a lower rate of using micro scissors (p = 0.32) and chandelier lighting system (p = 0.52) in the 27-gauge group.” p = 0.32 and 0.52 is not a trend. Indeed, the usage of 27 gauge instead of 25 gauge in theory should make surgeon use less intra ocular devices, but the author did not demonstrate it in this current study.

**Author response:** Though 27G vitrector can significantly reduce the use of micro forceps. We still cannot show the trend of less use in micro scissors and chandelier lighting system, though we did feel that the timing of using micro scissors is less. The possible explanation is that, first, the case number is not large enough, which could not show statistical significance, and second, though we still use micro scissors in 27-gauge system in some complicated cases, the total times in each surgery using micro scissor may still be less, however, we need another study to confirm this. In the revised manuscript, I will change the sentence of “trend” to “no significant trend” (**Lines 140-141**), instead, we will discuss why we did not show significantly less usage of micro scissors in this study (**Lines 184-188**).

**Response to Reviewer # 2:**

1. **Comment:** In the methods and materials, you’ve discussed the status of pre-operative treatment including Anti-VEGF, you have not mentioned the pre-operative treatment with laser pan photocoagulation PRP which usually shorten the time of surgery – if previously done- and also make the vitrectomy easier for some extent

**Author response:** Thank you for the reviewer point out the weak point of this study. In the revised manuscript, the information of previous PRP will be added. We will add the pre-operative PRP, intra-operative PRP spot number (**Lines 136-137, Table 1 and 2**), and thus, this PRP number cannot influence total operation time (**Lines 299-300, Line 308-309**).

2. **Comment:** Regarding post-operative BCVA improvement, how did you define the improvement especially in cases with combined PPV and cataract surgery?

**Author response:** There are only two cases had received simultaneous cataract surgery, without statistical significance. One of them is in the severity stage 4 of TRD, and cataract is not an important factor to influence the visual acuity improvement. The other one received cataract surgery due to intra-operative iatrogenic cataract, and this cannot influence the visual acuity improvement. We added the simultaneous cataract surgery (**Lines 138-139, Table 2**) and further discussed this. (**Lines 306-308**)

3. **Comment:** Have you noticed any significant difference in post vitrectomy cataract development between 25G and 27G ?

**Author response:** In the 27-gauge group, there were 10 cases receiving cataract surgery 6 months later or longer after, and 3 cases received cataract surgery at 4, 4, 5 months. While in the 25-gauge group, there were 15 cases receiving cataract surgery 6 months later or longer after, and 3 cases received cataract surgery at 1, 1, 3 months. There are no significant difference between 2 groups in cataract development. We will add time to develop cataract. (**Lines 315-317, Table3**)

4. **Comment:** As the cutter and the light pipe are thinner in 27G, have you noticed more bending of the instruments in 27G than that of 27G, or there was no significant difference?

**Author response:** We did notice there was more chance of instrument bending in the 27-gauge system when we initially use 27-gauge system. However, after a short learning curve, bending of instrument seldom happens. To avoid bending of instruments, you should not rotate the eye ball too much with the instrument as we used to do with 25- or 23-gauge system. To address the far peripheral lesions, use indentation, instead of rotating the eye ball.
